# Supplementary material for: Safety and efficacy study: Short-term application of radiofrequency ablation and stereotactic body radiotherapy for Barcelona Clinical Liver Cancer stage 0–B1 hepatocellular carcinoma
Source: PLoS One. 2021 Jan 5;16(1):e0245076. doi: 10.1371/journal.pone.0245076 (PMC7785216; doi:10.1371/journal.pone.0245076)
Supplement: S1 File — (DOCX) [file pone.0245076.s001.docx]

**The operation details of RFA were as follows.**

*Instruments and equipment*

RFA was performed under either grayscale US guidance (for HCC clearly detected by grayscale US) or contrast-enhanced US (CEUS) guidance (for US undetectable HCC). The LOGIQ E9 ultrasound system and a convex probe with a frequency of 1–6 MHz or a micro-convex probe with a frequency of 2–5 MHz were applied. To ablate all the tumors, RFA was performed using a 480 kHz generator (VIVA RF generator; STARmed, Gyeonggi, Korea), capable of producing a maximum power of 200 W and a specific size of 17-gauge internally cooled, adjustable RF electrode (Proteus; STARmed, Gyeonggi, Korea).

*Important parameter setting*

For CEUS guidance, in general, a low mechanical index (MI) mode (0.2–0.3) was used as the whole liver, which could be observed in real time, allows for repeated observations of the liver in a dynamic real-time manner because microbubbles stay static and undergo less destruction. Unfortunately, deep-located hepatic lesions might invisible with a low MI because of the attenuation of the US beam. If the lesion margin was indistinct (especially if the lesion was isoechogenic) on the grayscale US image and deep-located, high MI mode (0.8–1.2) and/or fusion imaging with enhanced CT/MRI were applied.

*Operation tips*

1)The lengths of the active tip of the electrodes were 5 mm, 1.0 cm, 1.5 cm, 2.0 cm or 3.0 cm. Each electrode was selected based on tumor size, tumor location, and operator preference. Briefly, for patients with a tumor diameter of 2 cm or smaller, an electrode with a 2 cm tip was inserted into the tumor and ablated at 20 W. For patients with a tumor diameter larger than 2 cm, an electrode with a 3-cm tip and ablation at 40 W was selected. For nodules with a relatively larger size (3–5 cm), the electrode was inserted at different sites.

2)In cases with hypervascular HCCs that exhibited hypervascular enhancement during the arterial phase and hypo-echoic enhancement during the post-vascular phase, we punctured the lesion during the post-vascular phase. In contrast, in cases with hypervascular HCCs that exhibited hypervascular enhancement during the arterial phase and iso-echoic enhancement during the postvascular phase, we punctured the lesion during the arterial phase of CEUS.

*Operation steps*

All patients received local anesthesia and analgesia before the procedure. In general, for lesion with big size, the first puncture of RFA targeted the center of the largest section of the lesion shown on the US images. Afterwards, the overlapping ablations were performed until the entire lesion was ablated. The ablation algorithm was based on elevations in tissue impedance. The mean duration of one ablation was 12 minutes, and the temperature of the ablated tissue was maintained above 60 ℃.

Immediately after ablation, the therapeutic response to RFA was evaluated by CEUS or fusion imaging to determine the adequacy of ablation, and whether additional ablation was needed. If sometimes the steam produced by radiofrequency ablation affects the visual field of ultrasound observation, wait for a moment for a clear field of vision before evaluating the adequacy of ablation. For RFA performed under the guidance of CEUS, post-operative CEUS examination was undertaken to see if the edge of the ablation area showed hypervascularity in AP. A complete ablation could be confirmed by no perfusion of contrast agent into the tumor as a whole, showing a “cavity” appearance with a distinct boundary. For RFA treatment guided by fusion imaging, the overlap function of fusion imaging was applied to observe whether the ablative area completely covered the lesion area shown on pre-operative enhanced MRI or CT images.

After ablation, the needle was retracted, maintaining its tip hot in order to prevent, by thermal coagulation, seeding or haemorrhage along the electrode track. Every procedure was aimed at obtaining a no less than 5 mm safety margin around the treated lesions.

**The operation details of SBRT were as follows.**

During free breathing of patients, Spiral, 4-phase, multidetector CT and/or dynamic contrast-enhanced MRI were conducted, and followed by fusion with a slow-scan CT scan (6–10 seconds per slice). The gross tumor volume (GTV), including enhanced tumor, was delineated with the slow-scan CT images. For the internal target volume, an internal margin (4–6 mm) was created around the clinical target volume (CTV) according to the respiratory movement of the diaphragm observed during fluoroscopy. For the planning target volume (PTV), individualized margins of 2 mm were applied around the internal target volume as a setup margin. Multiarc, dynamic conformal radiation was planned using a radiation treatment planning system (FOCUS XiO, version 4.2.0–4.3.3: Computerized Medical Systems, St Louis, Mo) and was performed using X-rays from a 6-MV linear accelerator (Clinac 2100C; Varian Medical Systems Inc, Palo Alto, Calif). Generally, SBRT with total doses of 35 Gy or 40 Gy were delivered in five fractions over 5–7 days.

For patients with Child-Pugh grade A–B grade, > 20% of the normal liver receiving > 20 Gy, and a total dose of 35 Gy was administered. For other patients, a total dose of 40 Gy were delivered. Notably, for the lesions which were close to the gastrointestinal tract, there was a policy that the dose to a 10-cc area of the gastrointestinal tract should be limited to <25 Gy. In this consideration, the treatment strategy was hypofractionated radiotherapy: a total dose of 36–45 Gy in 12–15 fractions over 16–21 days. Treatment was planned to enclose the planning target volume with a maximal dose of 60%–80% isodose line.
